# The mTORC2 Component Rictor Is Required for Load‐Induced Bone Formation in Late‐Stage Skeletal Cells

**DOI:** 10.1002/jbm4.10366

**Published:** 2020-06-18

**Authors:** Karl J Lewis, Xin Yi, Christian S Wright, Emily Z Pemberton, Whitney A Bullock, William R Thompson, Alexander G Robling

**Affiliations:** ^1^ Department of Anatomy & Cell Biology Indiana University School of Medicine Indianapolis IN USA; ^2^ Department of Physical Therapy Indiana University School of Health & Human Sciences Indianapolis IN USA; ^3^ Indiana Center for Musculoskeletal Health Indianapolis IN USA; ^4^ Department of Biomedical Engineering Indiana University–Purdue University at Indianapolis Indianapolis IN USA; ^5^ Richard L. Roudebush VA Medical Center Indianapolis IN USA

**Keywords:** ACTIN, MECHANOTRANSDUCTION, mTOR, OSTEOCYTES, RICTOR

## Abstract

Bone relies on mechanical cues to build and maintain tissue composition and architecture. Our understanding of bone cell mechanotransduction continues to evolve, with a few key signaling pathways emerging as vital. Wnt/β‐catenin, for example, is essential for proper anabolic response to mechanical stimulation. One key complex that regulates β‐catenin activity is the mammalian target of rapamycin complex 2 (mTORc2). mTORc2 is critical for actin cytoskeletal reorganization, an indispensable component in mechanotransduction in certain cell types. In this study, we probed the impact of the mTORc2 signaling pathway in osteocyte mechanotransduction by conditionally deleting the mTORc2 subunit Rictor in Dmp1‐expressing cells of C57BL/6 mice. Conditional deletion of the Rictor was achieved using the Dmp1–Cre driver to recombine Rictor floxed alleles. Rictor mutants exhibited a decrease in skeletal properties, as measured by DXA, μCT, and mechanical testing, compared with Cre‐negative floxed littermate controls. in vivo axial tibia loading conducted in adult mice revealed a deficiency in the osteogenic response to loading among Rictor mutants. Histological measurements of osteocyte morphology indicated fewer, shorter cell processes in Rictor mutants, which might explain the compromised response to mechanical stimulation. In summary, inhibition of the mTORc2 pathway in late osteoblasts/osteocytes leads to decreased bone mass and mechanically induced bone formation. © 2020 The Authors. *JBMR Plus* published by Wiley Periodicals, Inc. on behalf of American Society for Bone and Mineral Research.

Mechanical stimulation of bone tissue is a major determinant of skeletal mass, distribution, and strength.^1^ Enhanced loading (eg, vigorous exercise) presents a strong anabolic stimulus to resident bone cells. Although many cell types housed within bone tissue are mechanoresponsive, it is generally recognized that the osteocyte is the primary mechanosensory cell type for bone (see ref. 2^2^ for review). Mechanically stimulated osteocytes transduce physical stimuli into biochemical signals that ultimately reach the bone surface, where the effector cell populations (osteoblasts and osteoclasts) or their progenitors can be accessed and directed for appropriate spatiotemporal activity. The integrity of this intercellular transduction process relies on the fidelity of a multitude of complex signaling cascades within the osteocyte. For example, the Wnt/Lrp5 axis is crucial for mechanotransduction in osteocytes,^3^ as is autocrine/paracrine signaling involving prostaglandins,^4^, ^5^, ^6^ nitric oxide,^7^, ^8^ adenosine triphosphate (ATP),^9^, ^10^ and integrin/cytoskeletal components.^11^

Recently, the mammalian target of rapamycin (mTOR) pathway has been of particular interest in bone cell mechanotransduction. The mTOR subunit is a component of two distinct signal transduction pathways: mTOR complex 1 (mTORc1) and complex 2 (mTORc2). mTORc1 is minimally comprised of mTOR, raptor, and mLST8; is sensitive to rapamycin; and responds to nutrients/amino acids to control protein synthesis. Upstream and downstream components of mTORc1 are relatively well‐characterized (see ref. 12^12^ for review). The mTORc2 complex is minimally comprised of mTOR, Rictor, Sin1, and mLST8. Upstream and downstream components of mTORc2 are more poorly characterized than for mTORc1. Despite the paucity of data on mTORc2 biology, prior work demonstrates a critical role for mTORc2 in mechanically induced activation of β‐catenin, a crucial node in bone cell mechanotransduction.^13^ We found that mechanical stimulation of mesenchymal stem progenitor cells (MSPCs) recruits mTORc2 to focal adhesions where it orchestrates cytoskeletal reorganization, subsequent protein kinase B (AKT) activation, Gsk3β phosphorylation, and ultimately, β‐catenin survival and nuclear translocation.^14^, ^15^ Inhibition of the mTORc2 component Rictor disrupts mechanically induced cytoskeletal reorganization, AKT activation, and promotes β‐catenin degradation. In the MSPC population, mechanically induced mTORc2 signaling promotes differentiation toward the osteoblast lineage, while impairing adipogenic differentiation, whereas knockdown of Rictor enhances adipogenesis.

Manipulation of Rictor has proven to be an effective approach to modulating mTORc2 efficacy. Rictor global deletion in mice is lethal,^16^ but several in vivo investigations of conditional Rictor deletion in bone have revealed a crucial role for mTORc2 in achieving proper bone mass. For example, floxed Rictor alleles have been recombined early in the preosteoblast lineage (limb bud mesenchyme) using Prx1–Cre,^17^ later in the osteoprogenitor population using Osx–Cre,^18^ and in the mature osteoblast using Ocn–Cre.^19^ The experiments consistently found a low bone mass phenotype in Cre‐positive mice, with various other side effects including reduced osteoblast adhesion, developmental defects, and compromised mineralization. In this study, we investigated the skeletal consequences of late‐stage (Dmp1–Cre mediated) Rictor deletion, where floxed Rictor alleles were recombined in the late‐stage osteoblasts and osteocyte populations. Further, we probed the response to mechanical stimulation in mice with conditional Rictor loss using the in vivo tibial loading model. We found significantly impaired skeletal properties (bone mass, density, strength) in Rictor‐deficient mice, and also a significant reduction in load‐induced bone formation, which was accompanied by altered osteocyte morphological properties. Our results suggest that the mTORc2 complex in the osteocyte population is crucial for achieving peak bone mass, strength, and responsiveness to mechanical inputs.

## Materials and Methods

### Mice

Development of the conditional Rictor loss‐of‐function mouse model (Rictor^f/f^) has been reported elsewhere.^20^ Briefly, the targeted mice harbor loxP sequences flanking exon 11 of the endogenous Rictor gene. Development of the ^10kb^Dmp1–Cre transgenic mouse model has been reported elsewhere.^21^ Briefly, these mice harbor a transgene expressing Cre recombinase driven by a 9.6‐kb fragment of the mouse dentin matrix protein‐1 promoter. All mouse colonies were maintained on a C57BL/6 background. Cre‐mediated recombination of the floxed allele was assessed by real‐time PCR of purified osteocyte‐enriched genomic DNA (gDNA) extracted from cortical bone tissue at the distal humerus as described earlier.^22^ The forward primer was located within exon 11 (the floxed region) and the reverse primer was located 3′ to the distal loxP site. As an internal control, exon 12 (unmodified) was also amplified by PCR. For all experiments, Cre‐positive Rictor^f/f^ males were bred to Cre‐negative Rictor^f/f^ females to produce Cre‐positive and Cre‐negative pups, each of which was homozygous for the Rictor conditional allele. Mice were genotyped for the Rictor WT/flox alleles and Dmp1‐Cre transgene using PCR on tail‐tip DNA with primers provided in the original publications.^20^, ^21^ Both male and female littermate mice (group housed by sex and litter) were used in all experiments, and were given standard mouse chow (2018SX, Harlan‐Teklad, Indianapolis, IN, USA; 1% Ca; 0.65% P; 2.1 IU/g vitamin D3) and water *ad libitum*. All mouse procedures were performed in accordance with the IACUC guidelines and approvals.

### Dual‐energy x‐ray absorptiometry (DXA)

Collection of serial DXA measurements on live mice are described and validated elsewhere.^23^ Briefly, mice were anesthetized via inhalation of 2.5% isoflurane (IsoFlo; Abbott Laboratories, North Chicago, IL, USA) mixed with O_2_ (1.5 L/min) for approximately 8 min, including both induction and scanning. The mice were placed in prone position on a specimen tray within the scanner. Whole‐body scans were analyzed regionally using the Lunar region of interest (ROI) tools. The ROI for the spine included from the third (LV_3_) through fifth (LV_5_) lumbar vertebra. The ROI for the hindlimb included all skeletal tissue distal the acetabulum. The ROI for the whole body included all skeletal tissues caudal to the CV¬1/skull boundary. Serial scans were performed at 6, 9, 12, and 16 weeks of age. BMD was measured for each ROI scan.

### Micro‐computed tomography (μCT)

Formalin‐fixed left femora and fifth lumbar vertebra were scanned, reconstructed, and analyzed as previously described.^23^ Briefly, 10‐μm resolution, 50‐kV peak tube potential, and 151‐ms integration time were used to collect scans on a Scanco μCT‐35 tomographer (Scanco Medical AG, Brüttisellen, Switzerland). The distal 60% of each femur and the entire body of each vertebra were scanned. Standard parameters related to cancellous and cortical bone architecture were measured.^24^

### Mechanical properties

Parameters related to whole‐bone strength were measured using 3‐point bending tests on isolated femora as previously described.^25^ Briefly, each femur was loaded to failure in monotonic compression, during which force and displacement were collected every 0.01 s. From the force/displacement curves, ultimate force, stiffness, and energy to failure were calculated using standard equations.^26^

### In vivo tibial loading

The axial tibial compression model was applied to Cre‐positive and Cre‐negative mice as previously described.^27^ Briefly, 20 calibration mice were sacrificed at 17 weeks of age to collect strain measurements. The right hindlimb was disarticulated and frozen at −20°C until strain gage testing. A single‐element strain gage (EA‐06‐015DJ‐120; Vishay Precision Group, Malvern, PA, USA) was applied to the midshaft of tibia on the posterior surface (surface between tibia and fibula) and the microstrain:load ratio was measured for each sample using progressively increasing load applications, while simultaneously recording the voltage output from the load cell and strain gage. All tests were averaged within each genotype to determine the microstrain:load ratio within each sex and Cre status. A peak microstrain value of −2250 was chosen to be applied to all genotypes and this corresponded to peak loads of 8.7 N (Cre‐negative females), 8.0 N (Cre‐positive females), 9.4 N (Cre‐negative males), and 8.5 N (Cre‐positive males). At 16 weeks of age, 11 mice of each Cre status began the axial tibia loading protocol. Mice were anesthetized using isoflurane inhalation, and their right hindlimb (knee to foot) was loaded using sinusoidal (haversine) waveform (2 Hz, 180 cycles, no intercycle rest period) to the peak load determined by strain gauging (see above). Mice were given three bouts over a 5‐day period with a day of rest between each bout. Intraperitoneal injection of calcein was given 1 day after the final bout, followed by an i.p. injection of alizarin complexone 8 days later. Mice were sacrificed 12 days after the final bout. The right and left tibias were harvested and placed in 10% NBF for 2 days followed by storage in 70% ethanol at 4°C.

### Histological processing and histomorphometry

Tibias and femora were dehydrated in graded alcohols, cleared in xylene, and embedded in methylmethacrylate following standard protocols. To measure load‐induced bone formation, thick‐cut sections were cut from the tibia approximately 3 mm proximal to the tibiofibular junction and manually ground down to approximately 30 μm. Tibial diaphysis sections were mounted unstained to visualize and read the calcein and alizarin labels administered at 17 and 18 weeks. To measure cortical bone apposition during the growth phase, thick‐cut sections were taken from the midshaft femur and manually ground down to approximately 30 μm. Left femur diaphysis sections were mounted unstained to visualize and read the demeclocycline and calcein labels administered at 6 and 17 weeks. To measure cancellous bone formation parameters, distal left femur sections were cut approximately 5 μm in thickness in the coronal plane, using a motorized microtome (Leica Microsystems, Inc., Wetzlar, Germany) equipped with a tungsten carbide knife. Distal femur thin sections were mounted unstained to visualize and read the calcein and alizarin labels administered at 17 and 18 weeks, or stained for tartrate‐resistant acid phosphatase (TRAP)/methyl green or toluidine blue to visualize and enumerate osteoclasts and osteoblasts, respectively. Sections were imaged on a fluorescent microscope using filter sets that provide excitation and emission for the demeclocycline, calcein, and alizarin wavelengths, or using white transmitted light for the cell counts. Digital images were imported into ImagePro Express (Media Cybernetics, Inc., Gaithersburg, MD, USA), and the following histomorphometric measurements were recorded for the periosteal surface: total perimeter, single‐label perimeter (sL.Pm), double‐label perimeter (dL.Pm), interlabel thickness (Ir.L.Th), and total bone area and marrow area. The following results were calculated: mineral apposition rate (MAR = Ir.L.Th/8 day), mineralizing surface (MS/BS = (0.5 × sL.Pm + dL.Pm) / total perimeter × 100), and bone formation rate (BFR/BS = MAR × MS/BS × 3.65). Relative formation parameters for loading effects were calculated for each mouse by subtracting the nonloaded (left tibia) values from the loaded (right tibia) values. All measurements were collected such that the operator was blinded as to group identity.

### Osteocyte cell process imaging and quantification

Femurs from Cre‐negative and Cre‐positive mice were decalcified for 8 days in 10% EDTA, embedded in paraffin, and sectioned (5 μm) longitudinally. Sections were immersed in xylene (2 × 5 min), then a series of graded ethanols including 100%, 95%, and 70% for 5 min each, followed by two 5‐min washes with PBS. Sections were permeabilized with Triton X‐100 (0.2%) in PBS for 1 hour and washed with PBS (3 × 5 min). Blocking buffer, containing BSA (1%) and Triton X‐100 (0.05%) in PBS was added to the slides for 1 hour at room temperature. A solution containing Alexa488‐conjugated phalloidin (Invitrogen cat# A12379, Invitrogen, Carlsbad, CA, USA; 1:100) and 4′,6‐diamidino‐2‐phenylindole (DAPI; Thermo cat# 62248, Thermo Fisher Scientific, Waltham, MA, USA; 1:1,000), diluted in PBS containing BSA (1%), was added to the tissue sections and incubated in a dark, humidified chamber at 37°C for 2 hours. Sections were washed with PBS, followed by the addition of mounting media and a coverslip. Images of cortical osteocytes, stained with Alexa488‐phalloidin, were acquired with a Leica SP8 resonant confocal microscope (Leica, Buffalo Grove, IL, USA). Osteocyte images, from femurs of three separate mice per group (Cre‐negative and Cre‐positive), were taken at the midshaft of the femur using a ×40 water‐immersion lens. A sample size of three mice per group was selected based on prior work assessing osteocyte canalicular dimensions in vivo.^28^ Five regions of interest (9 K μm^2^) were examined per bone section from each mouse. To quantify process number and length, 95 osteocytes from Cre‐negative and 114 osteocytes from Cre‐positive mice were evaluated. Microscope parameters, including laser power, signal gain, and pinhole size, were kept consistent across all sections imaged. Osteocyte cell process length was measured using the Neurite Tracer plugin within ImageJ software (NIH, Bethesda, MD, USA; https://imagej.nih.gov/ij/). Cell processes were included in the analyses only if the entire cell body was visible within the image, and if the origin of the origin of the cell process at the cell body was discernable. In addition to the length of the cell processes, the number of processes per osteocyte and the osteocyte density were quantified.

### Statistics

Sample sizes were determined using power calculations based on BMD effect sizes from a previously published Rictor study.^19^ Statistical analyses were computed with JMP software (version 12.0; SAS Institute Inc, Cary, NC, USA). The time‐series DXA data were analyzed within sex using repeated measures ANOVA. Tomography and histological and biomechanical endpoints were analyzed within sex using unpaired *t* tests. Statistical significance was taken at *p* < 0.05. Two‐tailed distributions were used for all analyses. Data are presented as mean ± SEM. A minimum of nine animals was included in each group (*n* = 9 to 11/group), except for the osteocyte cell process measurements, where *n* = 3/group.

## Results

### Mice with loss‐of‐function Rictor alleles in Dmp1‐expressing cells have global and regional deficits in bone mass and formation

**Figure 1:**
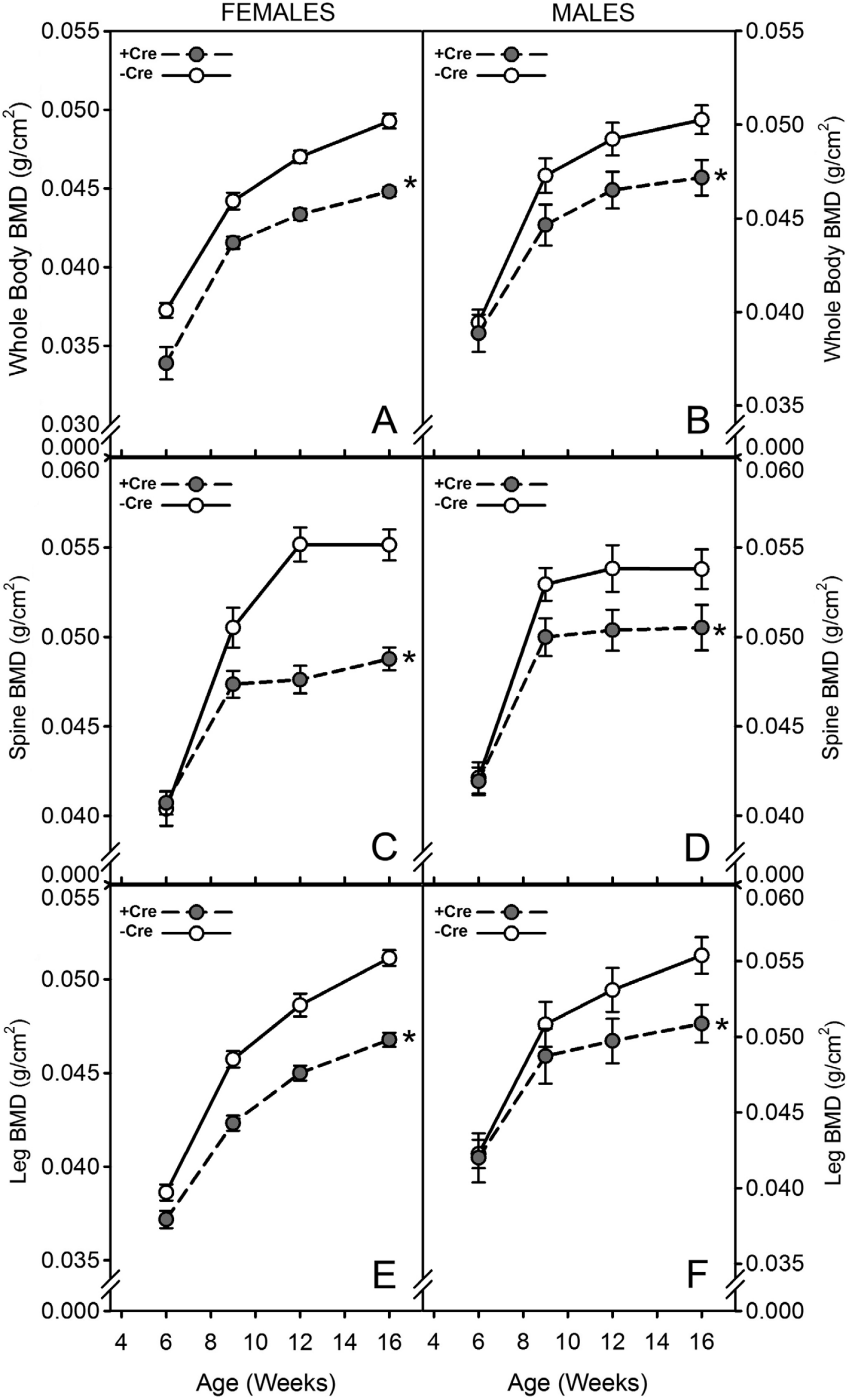
Serial in vivo DXA scans of Cre‐negative (solid lines) and ^10kb^Dmp1–Cre‐positive (broken lines) Rictor^f/f^ mice. Scans were collected every 3 to 4 weeks and analyzed for (*A*,*B*) whole‐body BMD, (*C*,*D*) lumbar spine BMD, and (*E*,*F*) BMD of the right hindlimb distal to the acetabulum. Panels *A*, *C*, and *E* display data from female mice; panels *B*, *D*, and *F* display data from male mice. The longitudinal data were tested for significance within sex, between Cre status, using repeated measures ANOVA. All comparisons are significant at *p* < 0.01. *n* = 9 to 12/group.

Previous studies addressing the skeletal effects of Rictor have focused on conditional deletion in cell types earlier in the osteoblast lineage, including limb bud mesenchymal cells (recombined with Prx1‐Cre), osteoprogenitors (recombined with Osx‐Cre), and mature osteoblasts (recombined with Ocn‐Cre).^17^, ^18^, ^19^ Here, we deleted Rictor later in the mesenchymal lineage—from late‐stage osteoblasts and osteocytes—using the Dmp1 promoter to drive Cre, and evaluated the effect on bone mass. Dmp1–Cre successfully recombined the allele in genomic DNA from osteocyte‐enriched lysates (Fig. S1). Serial whole‐body DXA scans were collected from all experimental mice intermittently from 6 to 16 weeks of age. For both males and females, Cre‐positive mice had significantly reduced (BMD for the whole‐body, spine, and hindlimb ROIs (Fig. 1 *A*‐1 *F*). Female Cre‐positive mice exhibited an approximately 6% to 13% deficit in BMD beyond 6 weeks of age (*p* < 0.01 for all ROIs), whereas males exhibited a 4% to 8% deficit in BMD beyond 6 weeks of age (*p* < 0.01 for all ROIs). Body weight was not different between Cre‐positive and Cre‐negative mice for either sex (Fig. S2). No adverse events were noted for any of the experiments.

**Figure 2:**
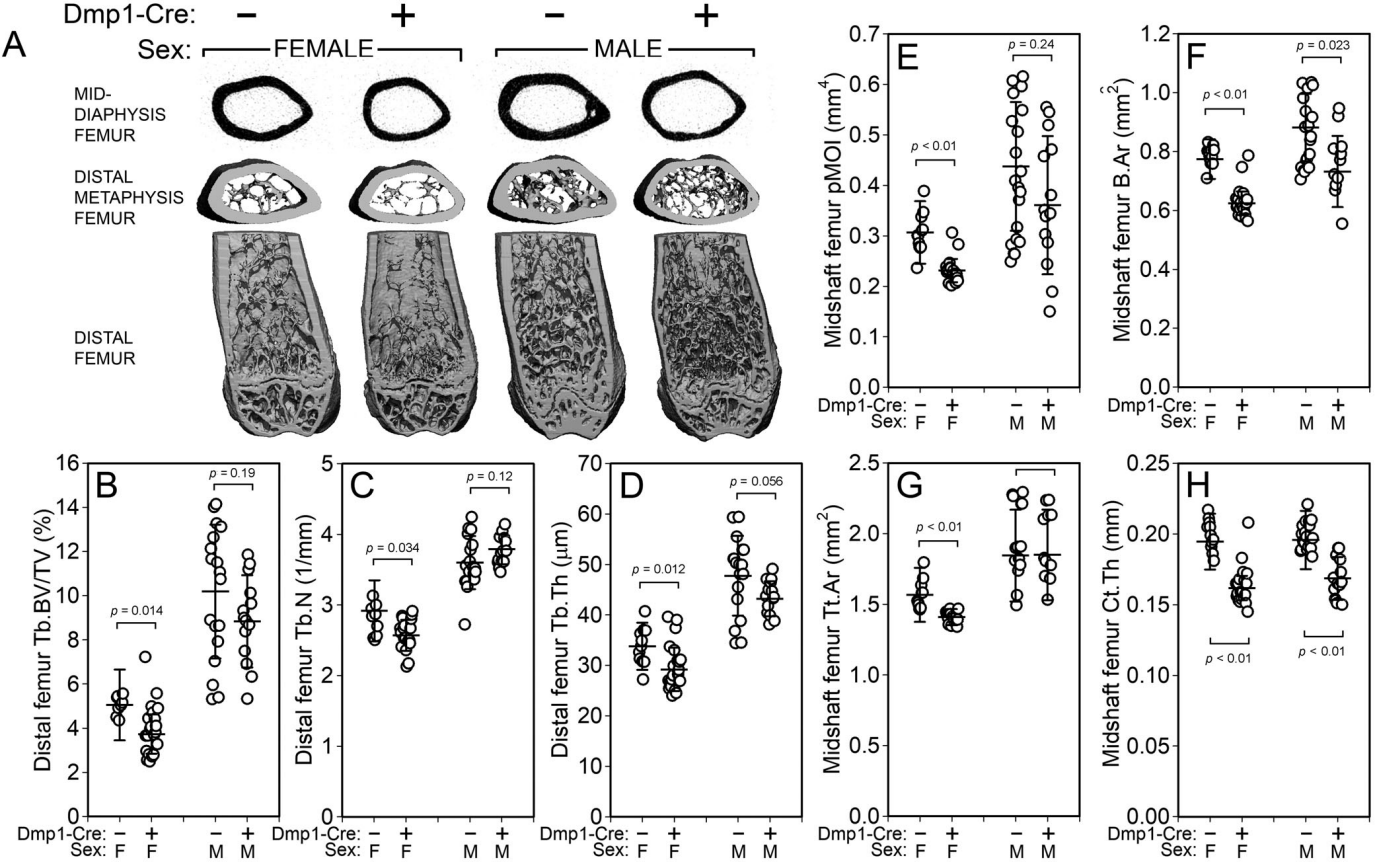
μCT‐derived measurement of the distal femur metaphyseal cancellous bone and midfemur cortical bone from Cre‐negative and ^10kb^Dmp1–Cre‐positive Rictor^f/f^ mice at 18 weeks of age. (*A*) Representative 3D reconstructions of (top row) the midshaft femur, (middle row) the distal metaphysis (proximal view), and (bottom row) the caudal half of the distal femur (the ventral half was digitally removed). Female mice are to the left and males to the right. Quantitative differences in femur (*B*) trabecular bone volume fraction (BV/TV), (*C*) trabecular number (Tb.N), and (*D*) trabecular thickness (Tb.Th), and (*E*) trabecular separation (Tb.Sp) are shown for female (left) and male (right) mice. Quantitative differences in femur cortical (*E*) bone polar moment of inertia (pMOI), (*F*) bone area (b.ar), (*G*) total area (Tt.Ar), and (*H*) cortical thickness (Ct.Th) are shown for female (left) and male (right) mice. Data were tested for significance within sex using unpaired *t* tests. *n* = 9 to 11/group.

To probe bone compartment‐specific effects of Rictor deficiency, the left femur of 18‐week‐old mice was scanned via μCT to assess distal femur cancellous properties and midshaft femur cortical properties. Bone volume fraction (BV/TV) was significantly reduced among female, but not male Cre‐positive mice, when compared with Cre‐negative littermates (Fig. 2 *A* and 2 *B*). Trabecular thickness was significantly reduced in Cre‐positive mice regardless of sex (Fig. 2 *D*), but trabecular number was significantly reduced by Rictor loss only in male mice (Fig. 2 *C*). Cortical bone properties were impaired in Cre‐positive mice, as indicated by significant reductions in midshaft femur bone area (B.Ar; approximately 20% decrease, *p* < 0.05; Fig. 2 *F*), and cortical thickness (Ct.Th; 14% to 17% decrease, *p* < 0.01; Fig. 2 *H*), with sex‐specific (female only) reductions in polar moment of inertia (25% reduction, *p* < 0.01; Fig. 2 *E*) and total area (10% reduction, *p* < 0.01; Fig. 2 *G*).

**Figure 3:**
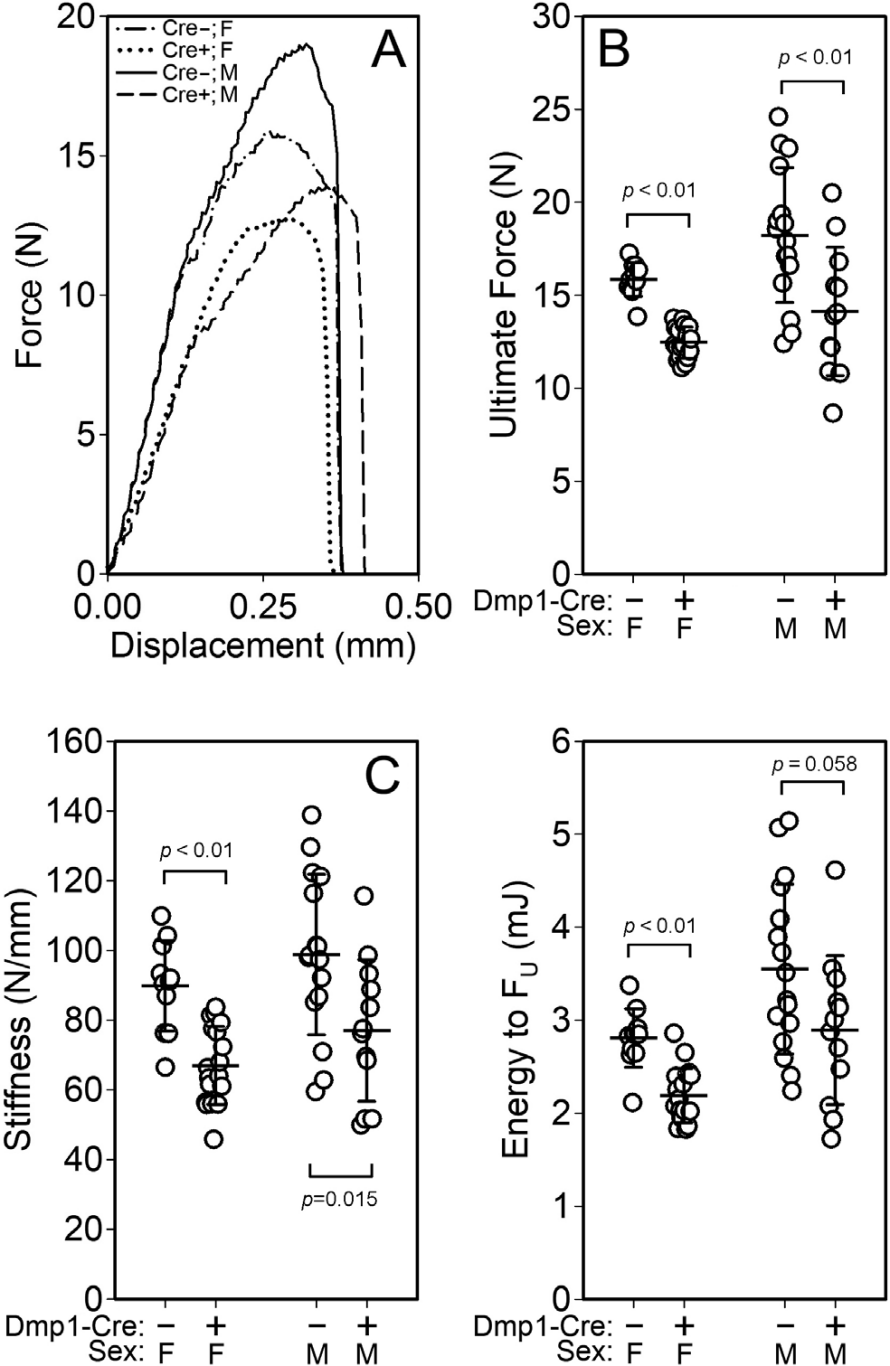
(*A*) Representative force–displacement curves from monotonic 3‐point bending tests to failure conducted on whole femora from female Cre‐negative and ^10kb^Dmp1‐Cre positive Rictor^f/f^ mice at 18 weeks of age. Quantification of (*B*) ultimate force, (*C*) stiffness, and (*D*) energy to ultimate force revealed significant deficiencies in the mechanical properties of Cre‐positive mice compared with controls. Data were tested for significance within sex using unpaired *t* tests. *n* = 9 to 11/group.

The deficit in cortical bone properties among mice with conditional inactivation of Rictor prompted us to determine whether there were associated deficits in mechanical/structural properties. Among femora from both male and female Cre‐positive mice, 3‐point bending tests (Fig. 3 *A*) revealed significantly reduced ultimate force (approximately 22% decrease for both sexes, *p* < 0.01; Fig. 3 *B*), stiffness (22% to 25% decrease, *p* < 0.05; Fig. 3 *C*), and energy absorption (18% to 22% decrease, *p* = 0.05; Fig. 3 *D*) compared with sex‐matched Cre‐negative mice. Bone formation rates (but not apposition rates) were reduced in the femoral cortex of Cre‐positive mice during the 6‐ to 17‐week growth phase, as revealed by an embedded 6‐week demeclocycline labels (Fig. S3). At 18 weeks of age, cancellous bone formation parameters were significantly reduced in Cre‐positive mice (Fig. S4), but osteoblast and osteoclast surfaces (Fig. S5) were not significantly affected.

### Mice with loss‐of‐function Rictor alleles in Dmp1‐expressing cells have compromised responsiveness to mechanical stimulation and altered osteocyte morphology

**Figure 4:**
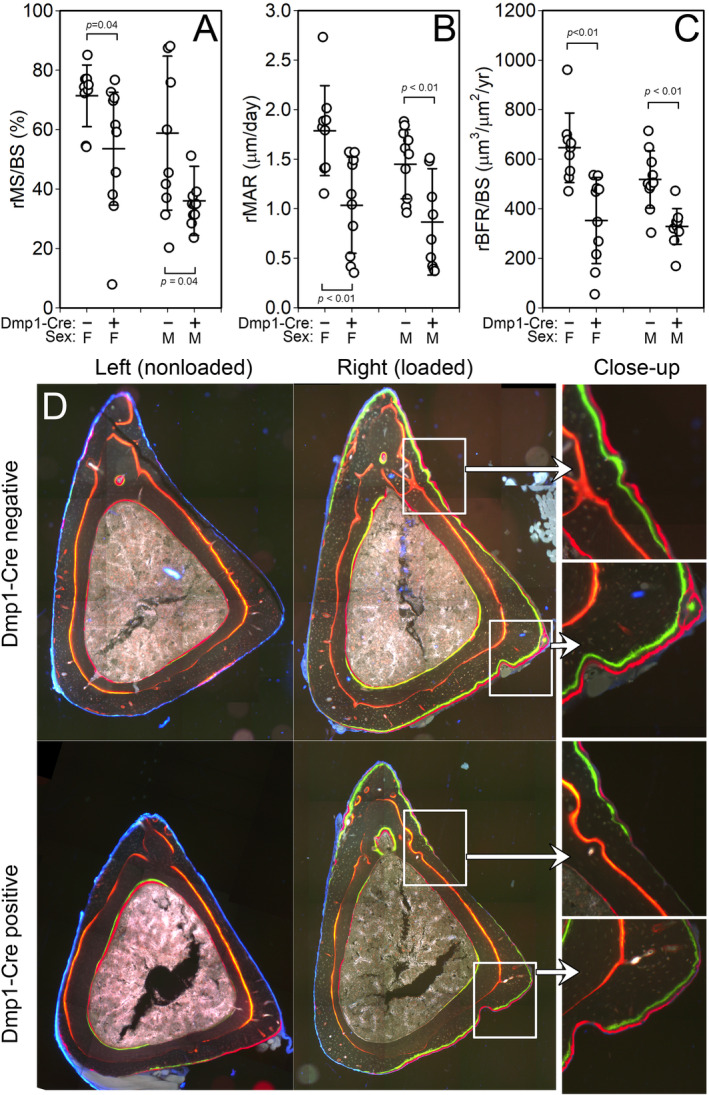
Axial tibial loading conducted in 16‐week‐old Cre‐negative and ^10kb^Dmp1–Cre‐positive Rictor^f/f^ mice. Strain‐matched peak forces were used to apply 1 week of loading to male and female mice. (*A*) Relative mineralizing surface, (*B*) relative mineral apposition rate (rMAR), and (*C*) relative bone formation rate per unit bone surface (rBFR/BS) on the periosteal surface were significantly suppressed in both female (left) and male (right) Cre‐positive mice compared with Cre‐negative controls. (*D*) Representative photomicrographs of fluorochrome‐labeled tibial diaphysis sections from left (nonloaded) and right (loaded) tibias, collected from female Cre‐positive and Cre‐negative mice. Data were tested for significance within sex using unpaired *t* tests. *n* = 7 to 10/group.

Previous work identified a critical role for the mTORc2 complex in regulating mechanical signal transduction in mesenchymal stem progenitor cells.^14^ Specifically, Rictor is necessary for mechanically induced actin stress fiber formation, a key process in bone mechanotransduction. To evaluate the role of mTORc2 in osteocyte‐selective mechanotransduction, we subjected 16‐week‐old conditional Rictor flox mice to anabolic mechanical loading in vivo using equivalent peak mechanical strains. In both Cre‐positive and Cre‐negative mice, significant load‐induced bone formation was detected (Fig. 4 *D*). However, Cre‐positive mice exhibited significantly suppressed relative mineralizing surface (25% to 28% decrease, *p* < 0.05; Fig. 4 *A*), MAR (28% to 42% decrease, *p* < 0.01; Fig. 4 *B*), and BFR (36–45% decrease, *p* < 0.01; Fig. 4 *C*) in response to axial tibial loading as compared with Cre‐negative controls.

**Figure 5:**
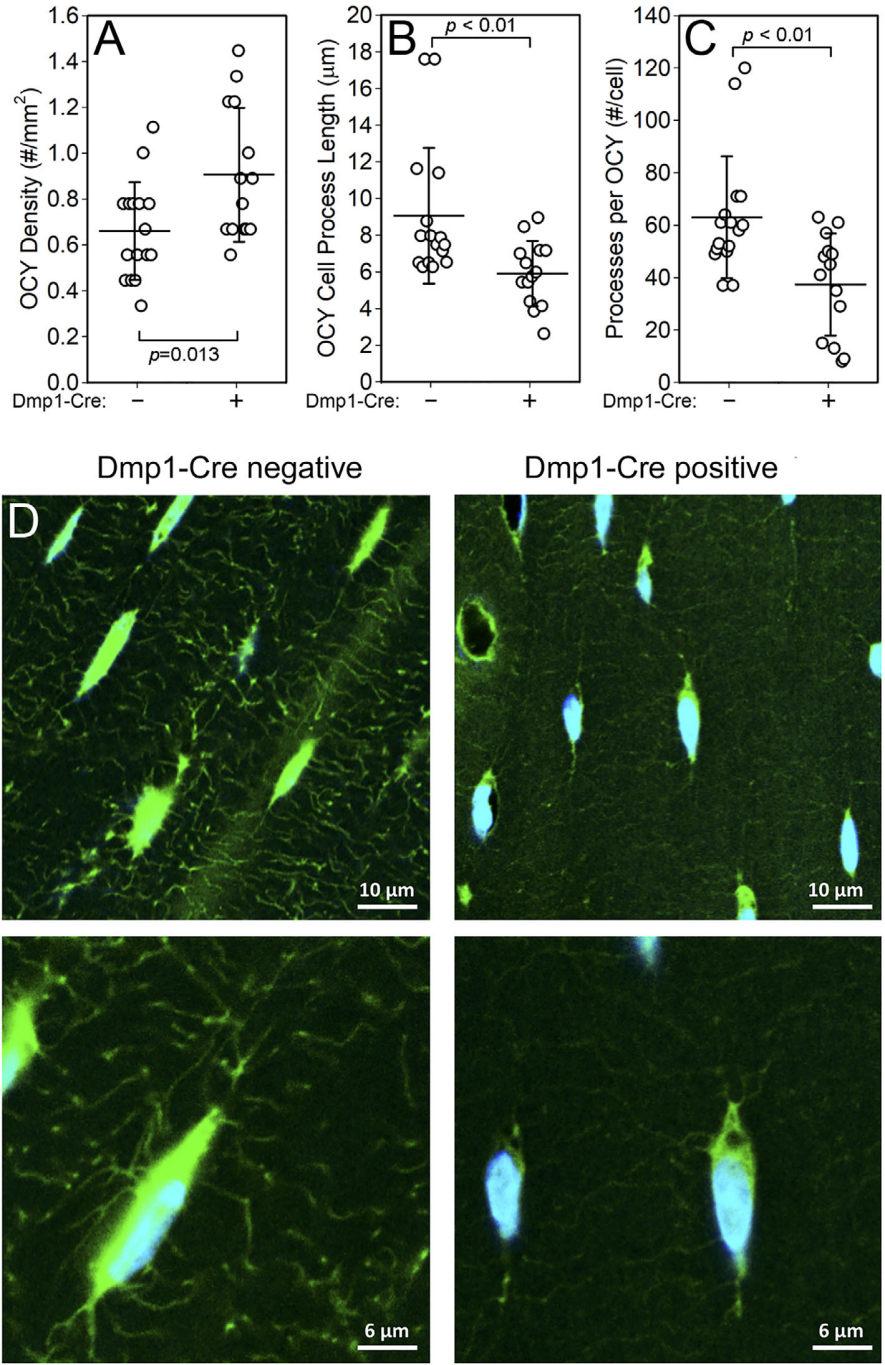
Phalloidin‐labeled cortical bone tissue sections from Cre‐negative and ^10kb^Dmp1‐Cre positive Rictor^f/f^ mice at 18 weeks of age. Quantitation of (*A*) the population density of osteocytes, (*B*) the mean length of the osteocyte cell process, and (*C*) the mean number of osteocyte cell processes per cell. (*D*) Representative photomicrographs of FITC‐phalloidin‐labeled cortical osteocytes collected from female Cre‐positive and Cre‐negative mice. Data were tested for significance within sex using unpaired *t* tests. *n* = 3 sections/group.

Several studies indicate that the actin‐rich cell processes of osteocytes are the most highly mechanosensitive subdomain of that cell type.^29^, ^30^ In light of our observation that osteocyte‐selective deletion of Rictor compromises load‐induced bone formation in vivo, we investigated whether Rictor inactivation in osteocytes alters the morphology or integrity of the cell processes; such an effect might explain the compromised response to skeletal loading in these mice. To this end, we cut cortical bone sections from Cre‐positive and Cre‐negative mice, stained them for filamentous actin using FITC‐labeled phalloidin, and measured cell process number and length. Cre‐positive mice exhibited a significant increase in the population density of osteocytes (37% increase, *p* < 0.05) and a decrease in both the number (26% decrease, *p* < 0.01) and average length (31% decrease, *p* < 0.01) of cortical osteocyte cell processes (Fig. 5 *A*‐5 *D*).

## Discussion

Our goal in this study was to investigate whether the mTORc2 complex plays a significant role in bone homeostasis and in the anabolic response to mechanical loading, when manipulated very late in the mesenchymal lineage (the osteoblast/late‐stage osteocyte population). Our inquiry was fueled by the published observations indicating that deletion of Rictor—a key member of the mTORc2 complex—earlier in the MSC/osteoblast lineage (ie, during Prx1 expression) resulted in a significant osteopenic phenotype. We found a strong effect of Rictor deletion from ^10kb^Dmp1‐expressing cells in both cortical and trabecular bone, though the effect was more consistent in the cortical compartment. The cortical deficiency for Rictor deletion in our study (Dmp1–Cre) was similar in magnitude to that reported by others using Prx1–Cre and Ocn–Cre, for comparable endpoints such as cortical thickness and cortical bone volume.^17^, ^19^ Considering all Cre driver lines together, the collective results suggest that much of the phenotype in early‐and mid‐stage deletion might be caused by effects that occur in the late‐stage cell populations.

Our second goal was to evaluate the anabolic response to skeletal loading in the absence of Rictor among ^10kb^Dmp1‐expressing cells. Our rationale for this experiment was based on previous observations in cultured MSPCs, whereby mechanically induced actin reorganization, AKT activation, and β‐catenin translocation were dependent on functional Rictor/mTORc2.^14^, ^15^ Tibia loading in 16‐week‐old Dmp1–Cre positive mice revealed a significant reduction in load‐induced bone formation parameters among both male and female mice. The magnitude of the deficiency in the mutants was similar to that reported for Prx1–Cre‐driven mutants,^17^ suggesting that most of the compromised loading effects in early‐stage deletion might be caused by effects that occur in the late‐stage cell populations (ie, osteocytes).

The mechanotransduction deficiency observed in Dmp1–Cre‐positive Rictor flox mice prompted us to investigate whether the osteocyte cell processes, which are actin‐rich structures,^31^ might exhibit impaired morphology. Osteocyte number was significantly increased by Rictor deletion, an observation that is difficult to rectify given the impaired mechanosensitivity. However, it is possible that the increase in osteocyte population density might reflect a feedback mechanism during growth in mutant mice to restore/improve the suppressed mechanosensitivity by increasing the number of sensor cells. We did observe a significant reduction in the number and length of osteocyte cell processes in mutant mice, as assessed by F‐actin stained cortical bone tissue sections. The actin measurements were conducted in light of the known effects of mTORc2 on actin cytoskeleton reorganization. As the osteocyte actin cytoskeleton is purported to modulate mechanosensitivity in both computational^32^ and experimental^33^, ^34^ models, the deficit in mechanotransduction induced by Rictor mutation is consistent with our measurements, indicating that cytoskeletal integrity is disrupted. However, we cannot rule out the possibility that Rictor controls mechanotransduction by other, yet undescribed, signal transduction mechanisms. As the biology of Rictor/mTORc2 becomes clearer, more‐refined insights into the mechanisms of action for Rictor in controlling basic bone homeostasis, as well as mechanotransduction, will likely emerge.

There are several limitations to the experiments reported herein. The ^10kb^Dmp1–Cre model has recently come under increased scrutiny based on observations showing Cre reporter allele recombination in tissues outside of bone (eg, muscle, perivascular marrow stromal cells, brain, and others).^35^ Therefore, the effects we observed in Rictor mutants could be influenced by altered signaling in other tissues. Second, our observations on osteocyte cell processes relied on actin staining to detect the processes. It is possible that the cell processes were as long and numerous in mutant mice, and merely the actin substructure was deficient. Either way, the deficit in actin staining within the osteocyte population is a likely cause for the compromised mechanotransduction observed.

In summary, mice with Rictor deficiency in the Dmp1‐expressing cells exhibit significantly reduced bone mass and strength. Moreover, load‐induced bone formation is compromised, supporting a role for mTORc2 activity in that process. If clinical use of mTORc2 inhibitors become a therapeutic option for certain types of cancers,^36^ concerns over concomitant bone loss and loss of mechanosensitivity are justified.

## Disclosures

All authors state that they have no conflicts of interest.

## Supporting information


**Figure S1** (Top) schematic showing the floxed Rictor allele (exons 11 is floxed), including the location of the primer pairs (red arrows) used to amplify in and around exons 11 and 12 to assess recombination. (Bottom) Ratio of intact Rictor at exon 11 to intact Rictor at exon 12 in cortical bone samples from Cre‐positive and Cre‐negative mice; n = 2‐4/group.

**Figure S2** Body mass in Cre‐negative (solid lines) and ^10kb^Dmp1‐Cre positive (broken lines) Rictor^f/f^ mice. Body mass was measured every 3–4 wks in both (A) female and (B) male mice. The longitudinal weight data were tested for significance using rmANOVA, For both sexes, *p* = 0.4–0.7*. n* = 9‐11/group.

**Figure S3** Periosteal bone formation parameters measured at the femoral midshaft of 18 wk‐old mice, using a pair of labels (demeclocycline label [orange] given at 6 wks of age and calcein [green] label given at 17 wks) to capture bone formation during the growth phase. Cortical bone formation rate (Ct.BFR/BS) but not mineral apposition rate (Ct.MAR) were significantly reduced in Cre‐positive mice. Note the distance between the orange and green periosteal labels (indicated by pink doubleheaded arrow). *n* = 9‐10/group.

**Figure S4** Distal femur cancellous bone formation parameters measured at the femoral midshaft of 18 wk‐old mice, using a pair of labels (calcein [green] label given at 17 wks and alizarin complexone [red] given at 18 wks) to capture bone formation in the secondary spongiosa. Trabecular mineralizing surface (Tb.MS/BS) and bone formation rate (Tb.BFR/BS), but not mineral apposition rate (Tb.MAR) were significantly reduced in Cre‐positive mice. *n* = 9‐10/group.

**Figure S5** Osteoblast and osteoclast surfaces were measured in the distal femur cancellous compartment of 18 wk old mice. The lower panels show Trap/methyl‐green stained sections used to enumerate osteoclast populations. *n* = 5‐7/group.
