# Supplementary figures and images for: The mTORC2 Component Rictor Is Required for Load‐Induced Bone Formation in Late‐Stage Skeletal Cells
Source: JBMR Plus. 2020 Jun 18;4(7):e10366. doi: 10.1002/jbm4.10366 (PMC7340445; doi:10.1002/jbm4.10366)

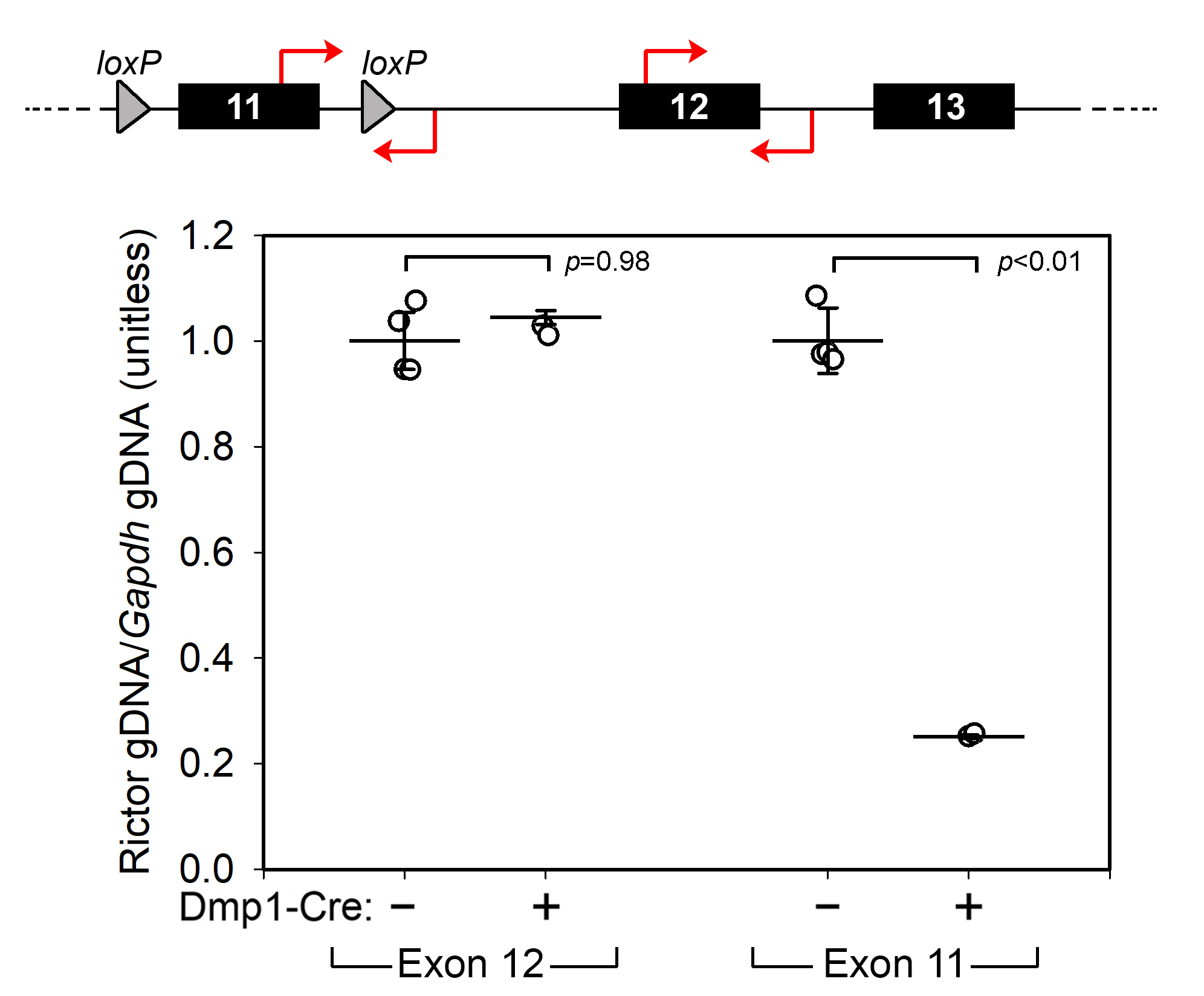

Supplement: Supplementary file 1 — Figure S1 (Top) schematic showing the floxed Rictor allele (exons 11 is floxed), including the location of the primer pairs (red arrows) used to amplify in and around exons 11 and 12 to assess recombination. (Bottom) Ratio of intact Rictor at exon 11 to intact Rictor at exon 12 in cortical bone samples from Cre‐positive and Cre‐negative mice; n = 2‐4/group. [file JBM4-4-e10366-s001.tif]

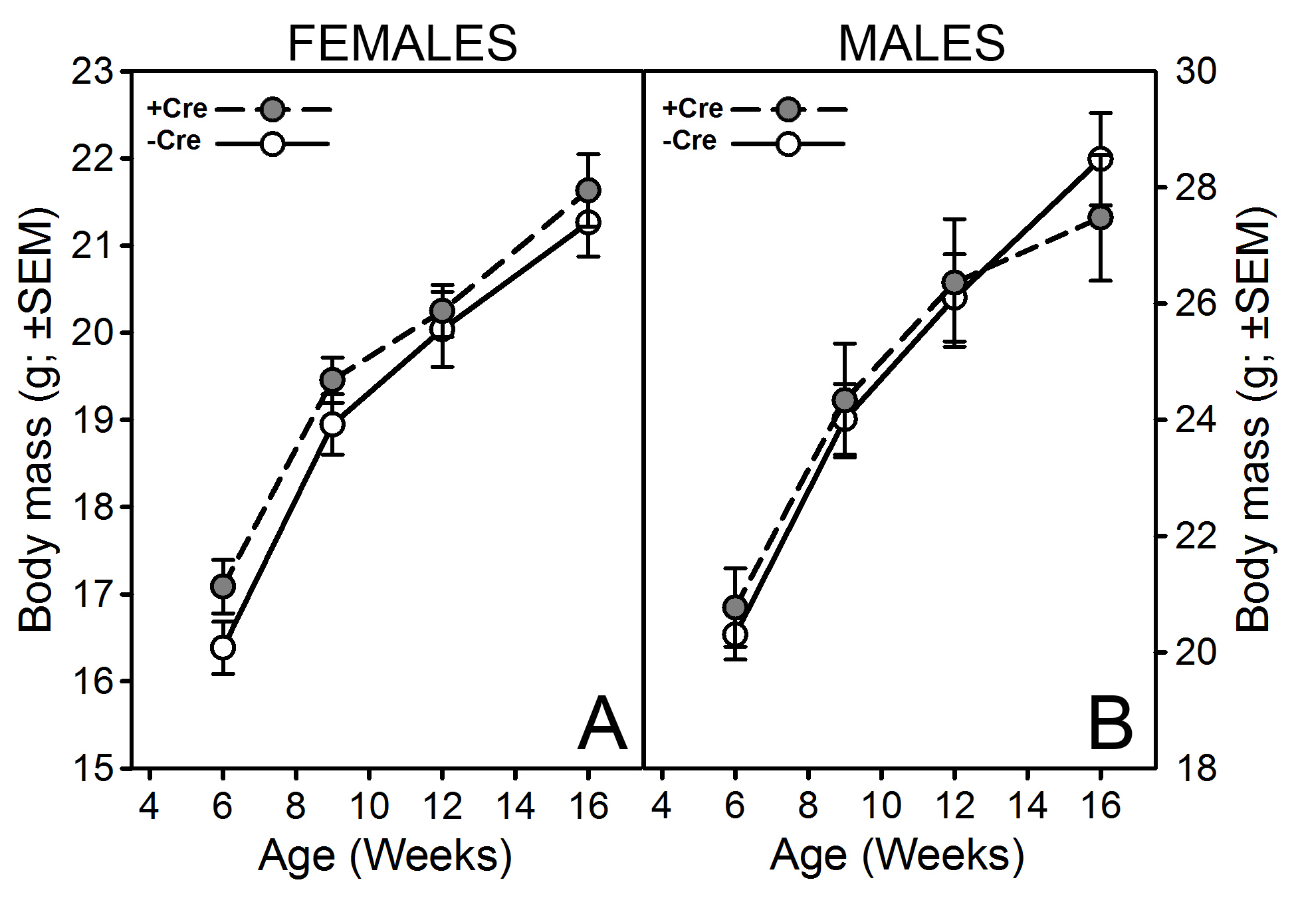

Supplement: Supplementary file 2 — Figure S2 Body mass in Cre‐negative (solid lines) and 10kbDmp1‐Cre positive (broken lines) Rictorf/f mice. Body mass was measured every 3–4 wks in both (A) female and (B) male mice. The longitudinal weight data were tested for significance using rmANOVA, For both sexes, p = 0.4–0.7. n = 9‐11/group. [file JBM4-4-e10366-s002.tif]

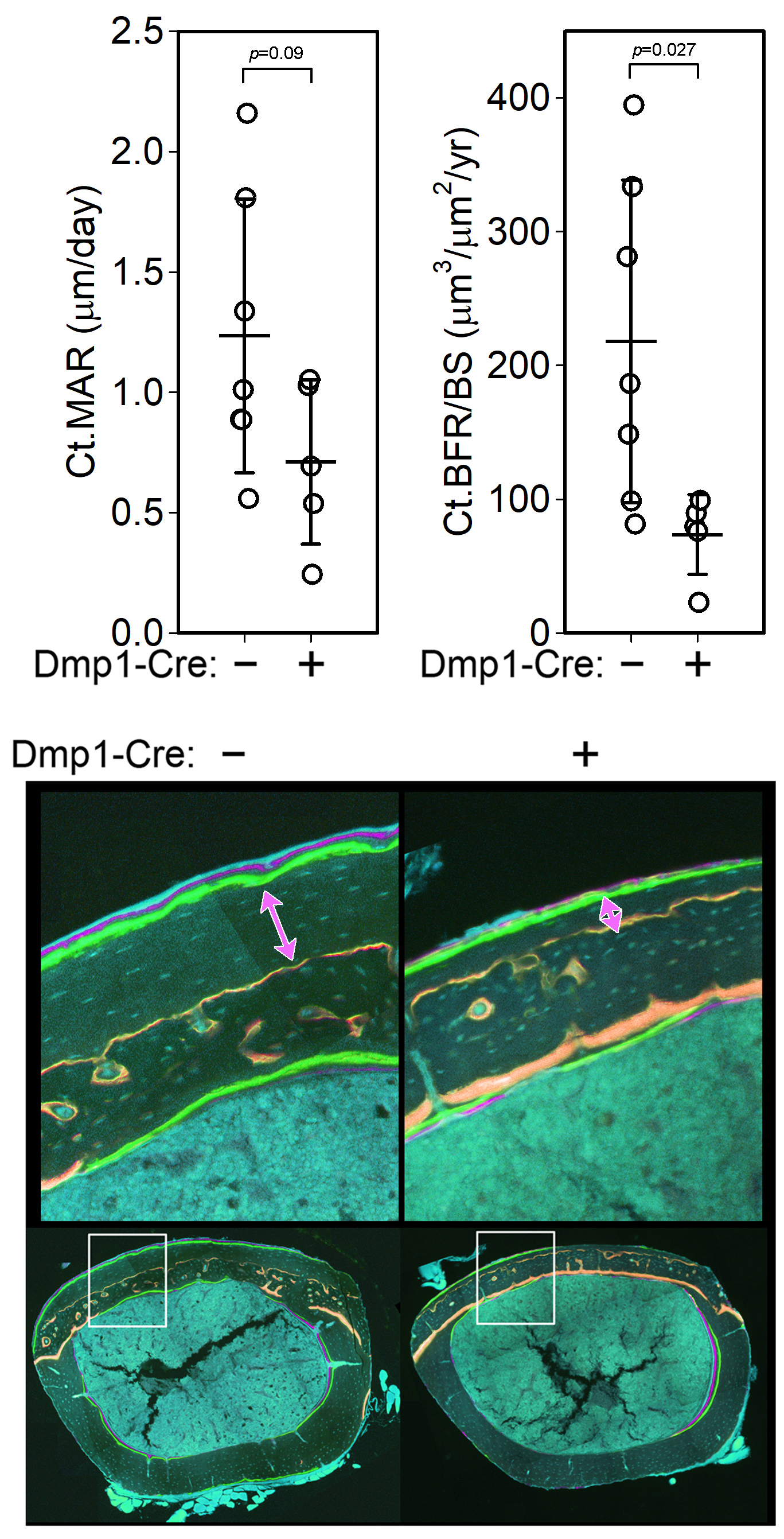

Supplement: Supplementary file 3 — Figure S3 Periosteal bone formation parameters measured at the femoral midshaft of 18 wk‐old mice, using a pair of labels (demeclocycline label [orange] given at 6 wks of age and calcein [green] label given at 17 wks) to capture bone formation during the growth phase. Cortical bone formation rate (Ct.BFR/BS) but not mineral apposition rate (Ct.MAR) were significantly reduced in Cre‐positive mice. Note the distance between the orange and green periosteal labels (indicated by pink doubleheaded arrow). n = 9‐10/group. [file JBM4-4-e10366-s003.tif]

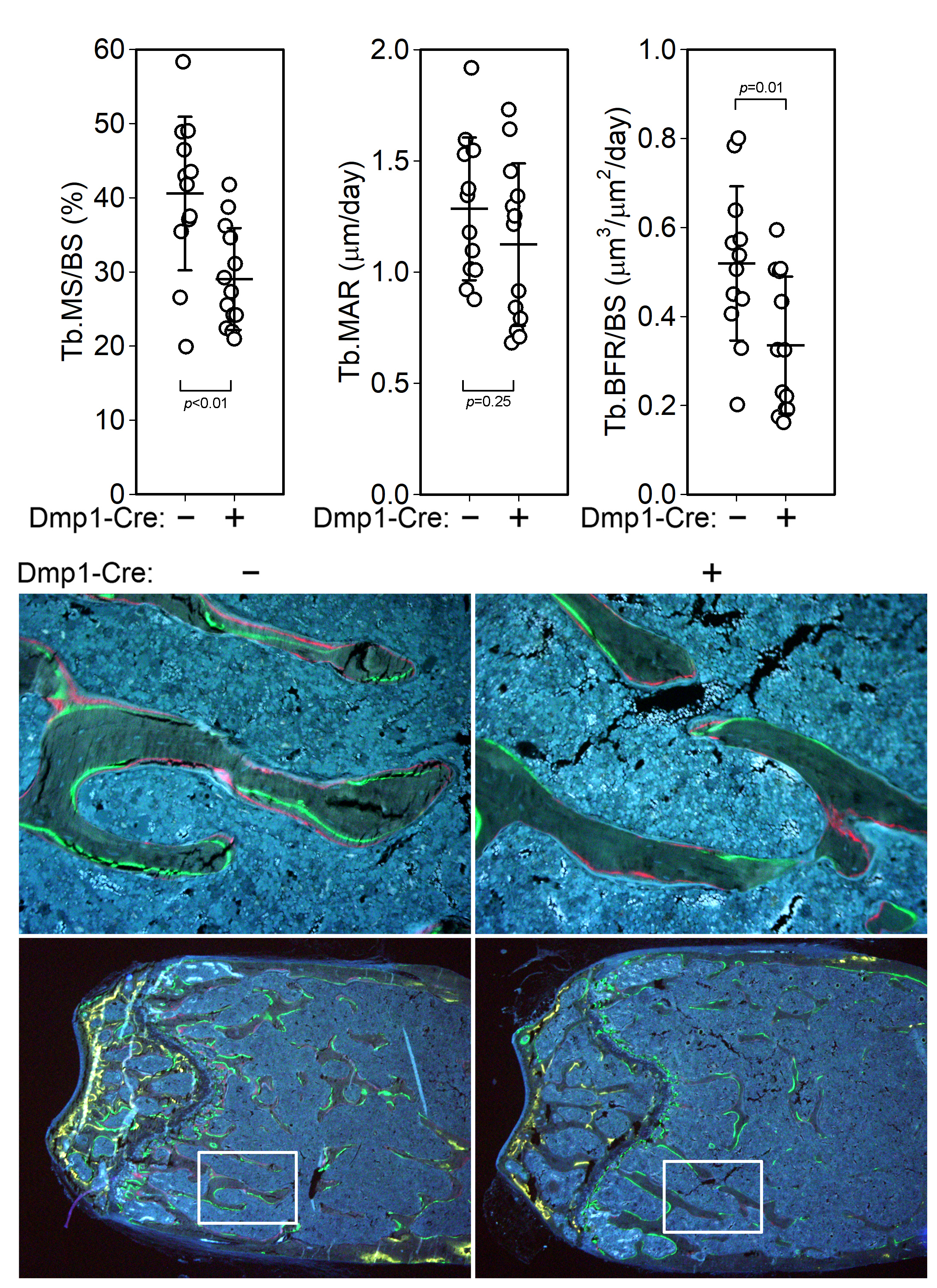

Supplement: Supplementary file 4 — Figure S4 Distal femur cancellous bone formation parameters measured at the femoral midshaft of 18 wk‐old mice, using a pair of labels (calcein [green] label given at 17 wks and alizarin complexone [red] given at 18 wks) to capture bone formation in the secondary spongiosa. Trabecular mineralizing surface (Tb.MS/BS) and bone formation rate (Tb.BFR/BS), but not mineral apposition rate (Tb.MAR) were significantly reduced in Cre‐positive mice. n = 9‐10/group. [file JBM4-4-e10366-s004.tif]

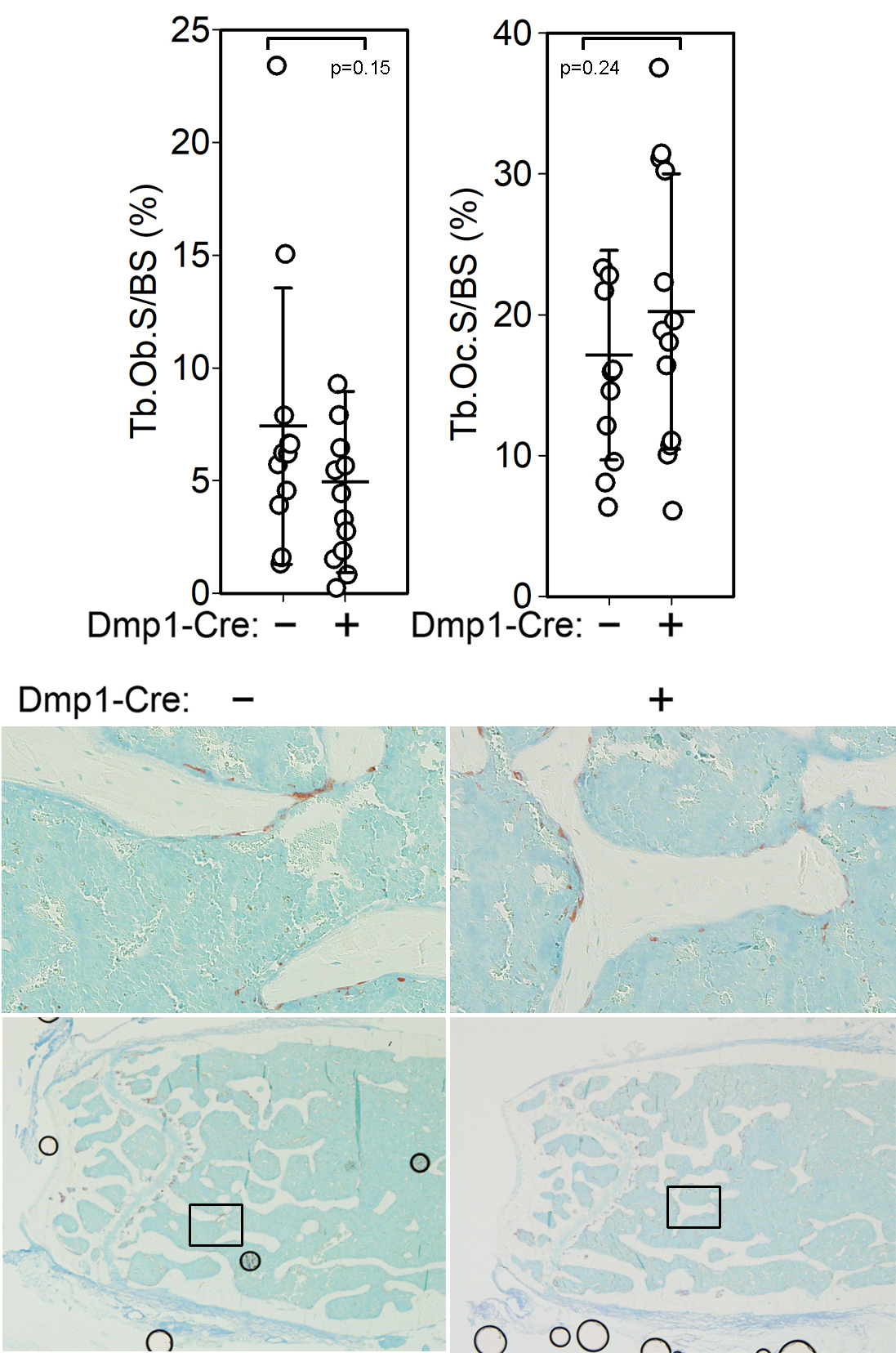

Supplement: Supplementary file 5 — Figure S5 Osteoblast and osteoclast surfaces were measured in the distal femur cancellous compartment of 18 wk old mice. The lower panels show Trap/methyl‐green stained sections used to enumerate osteoclast populations. n = 5‐7/group. [file JBM4-4-e10366-s005.tif]
